# Quercetin sensitizes glioblastoma to t-AUCB by dual inhibition of Hsp27 and COX-2 in vitro and in vivo

**DOI:** 10.1186/s13046-016-0331-1

**Published:** 2016-04-02

**Authors:** Junyang Li, Chao Tang, Liwen Li, Rujun Li, Youwu Fan

**Affiliations:** Department of Neurosurgery, Jinling Hospital, School of Medicine, Nanjing University, 305 East Zhongshan Road, Nanjing City, 210002 Jiangsu Province China; Department of Neurosurgery, Second Affiliated Hospital of Soochow University, 1055 Sanxiang Road, Suzhou, 215004 China

**Keywords:** Glioma, Soluble epoxide hydrolase, Heat shock protein 27, Cyclooxygenase 2, Inhibitor

## Abstract

**Background:**

Evidences indicate that inflammatory process plays pivotal role in tumor disease. Soluble epoxide hydrolase inhibitors (sEHIs) have been shown to participate in anti-inflammation and tumorigenesis by protecting epoxyeicosatrienoic acids (EETs). Although we have previously revealed some effects of t-AUCB on glioma in vitro, further investigations are needed to demonstrate its effects on glioblastoma growth in vivo and how to strengthen its antitumor effect.

**Methods:**

CCK-8 kit was used to test cell growth. Cell migration capacity was performed by wound healing assays. Transwell assay was used to test cell invasion potency. Cell-cycle analysis and cell apoptosis was performed by flow cytometry. The activity of caspase-3 in cells was measured using caspase-3 activity assay kits. Total RNA was extracted from cells lysated by TRIzol reagent. qRT-PCR was performed by ABI 7500 fast RT- PCR system. Lipofectamine RNAiMAX Transfection Reagent (Invitrogen) was used for siRNA transfection. Western blootting was used to test protein expression. Tumor cell xenograft mouse models were used for in vivo study. The SPSS version 17.0 software was applied for statistical analysis.

**Results:**

Our data shown that t-AUCB inhibits cell proliferation, migration and invasion and induces cell cycle G1 phase arrest in vitro but induces no cell apoptosis; increased Hsp27 activation and following COX-2 overexpression confer resistance to t-AUCB treatment in glioblastoma both in vitro and in vivo; quercetin sensitizes glioblastoma to t-AUCB by dual inhibition of Hsp27 and COX-2 in vitro and in vivo.

**Conclusions:**

These results indicate that combination of t-AUCB and quercetin may be a potential approach to treating glioblastoma.

## Background

Glioblastoma is the most common primary malignant tumor of the central nervous system in adults, which is highly aggressive and neurologically destructive. Despite the advances in surgery, radiotherapy and chemotherapy, survival time for patients with glioblastoma has remained at less than one year, not to mention the patients’ pain and heavy economic burden [1–5]. In view of the impossibility of real total resection of glioblastoma in surgery, and the serious side effects and the limited accessibility of radiotherapy, we suggest developing more efficient agent or combination of agents with great therapeutic effects and fewer side effects to treat glioblastoma or apply as postoperative adjuvant via circulatory system.

Recently, inflammation has been widely studied in malignant tumors and considered to participate in networks of activated signaling cascades, transcription factors and their coordinated interactions and promote tumorigenesis [6–8]. It could be effective therapy against malignant tumors to inhibit inflammation and then target inflammation-mediated transcription-factor interplay and signaling pathways [9]. Epoxyeicosatrienoic acids (EETs), a metabolite converted from arachidonic acid (ARA) by cytochrome P450 (CYP) epoxygenases, have been reported as mediators with antihypertensive, anti-inflammatory, analgesic, and cardioprotective effects [10]. EETs are easily to be hydrolyzed in vivo by soluble epoxide hydrolase (sEH) to form it’s less active or inactive metabolite dihydroxyeicosatrienoic acids (DHETs). Thus, various pharmacological inhibitors of sEH (sEHIs) have been developed to stabilize endogenous EETs and exert therapeutic effects [11]. Several studies have demonstrated that sEH play critical roles in angiogenesis and tumorigenesis, indicating the antitumor effects of sEHIs [12–14].

Our previous study has determined that t-AUCB, an improved sEHi synthesized and kindly provided by Prof. Hammock and his team, inhibits human glioblastoma cell growth, although cells then acquire apoptosis-resistance to t-AUCB via Hsp27 activation [15]. Considering the well proved antihypertensive, anti-inflammatory and analgesic effects of sEHIs which may greatly alleviate the pain of the patients, we suggest sEHIs may be a potential agent for glioblastoma treatment and worth further study. Recently, Prof. Hammock and his team demonstrated that a combination of COX-2 inhibitor and sEH inhibitor (t-AUCB) synergistically inhibits primary tumor growth. They also developed a COX-2/sEH dual inhibitor, PTUPB, which significantly suppresses primary tumor growth and metastasis [16]. In present study, we study the effects and interactions of Hsp27 inhibitor, quercetin, and t-AUCB on glioblastoma cells, and demonstrate that combination of quercetin and t-AUCB synergistically inhibits glioblastoma growth in vitro and in vivo. We also unexpectedly reveal that quercetin suppress COX-2 expression by Hsp27 inhibition and act as both COX-2 and Hsp27 inhibitor.

## Methods

### Regents

**Fig. 1:**
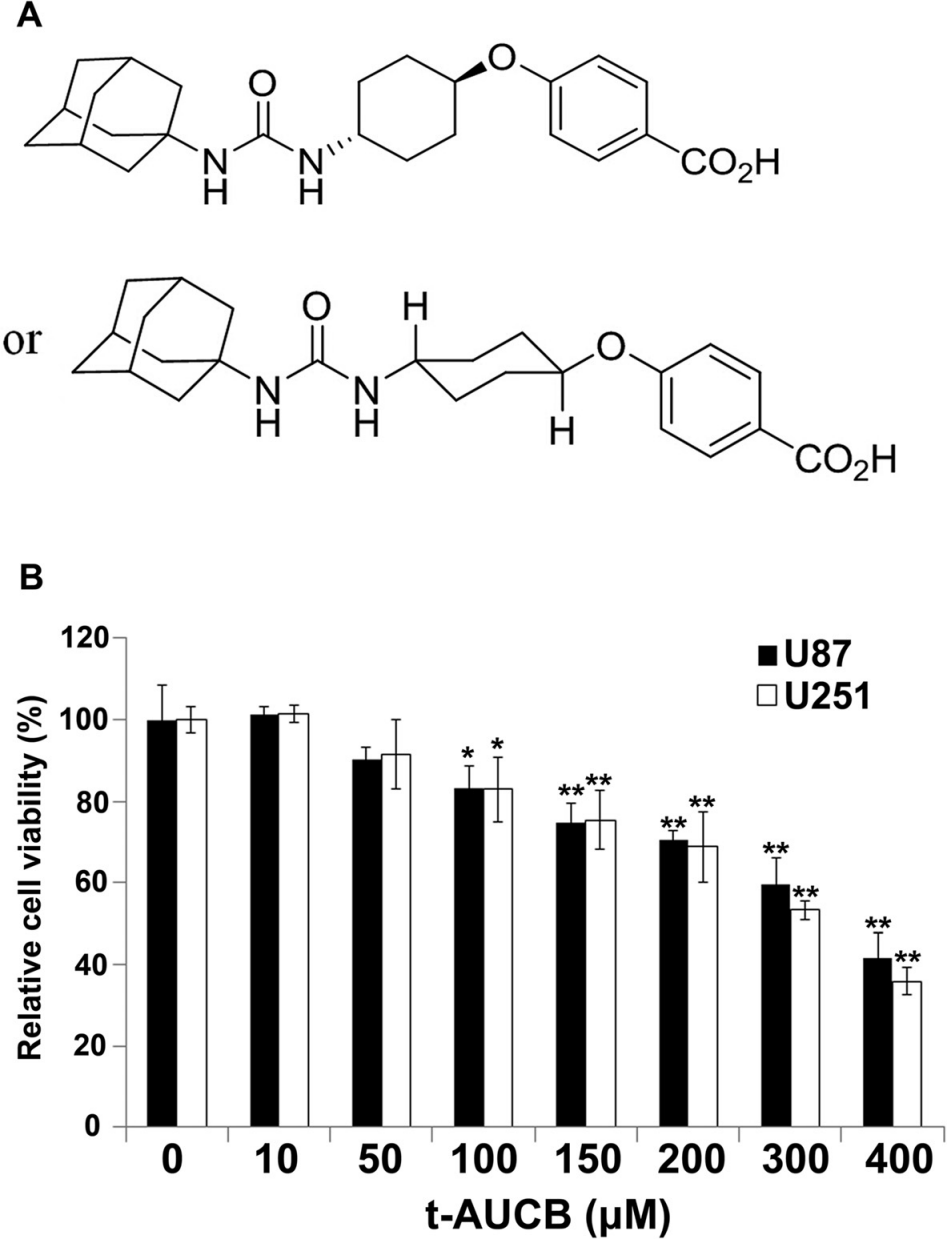
t-AUCB inhibits cell proliferation. **a**: The chemical constitution of t-AUCB. **b**: The percentage of OD value in t-AUCB treated cells compare with control represent the relative cell viability (%) in CCK-8 assay. U87 and U251 cells were treated with vehicle control (0 μM), 10 μM, 50 μM, 100 μM, 150 μM, 200 μM, 300 μM or 400 μM t-AUCB for 48 h. t-AUCB suppresses cell proliferation in a concentration-dependent manner since 100 μM (*P < 0.05). 150 μM to 400 μM t-AUCB causes more efficient cell growth inhibition (**P < 0.01)

The sEH inhibitor t-AUCB was granted from Professor Bruce D. Hammock (Department of Entomology and UCD Cancer Research Center, University of California, Davis, CA, USA) [17], and the chemical constitution of t-AUCB was shown in Fig. 1a. The p38 MAPK inhibitor SB203580 and Hsp27 inhibitor quercetin were both purchased from Sigma-Aldrich (St. Louis, MO, USA). All of agents were dissolved in dimethyl sulfoxide (DMSO). The concentration which was never exceeded 0.1 % (v/v) was diluted in serum-supplemented medium immediately before use. For cell growth assays, cells were treated with t-AUCB in different final concentrations of 0, 10, 50, 100, 150, 200, 300 or 400 μM, and quercetin in final concentrations of 0, 5, 15, 30 or 60 μM. For p38 MAPK inhibition, cells were treated with SB203580 in 20 μM as we have described previously [15]. For Hsp27 inhibition, cells were treated with quercetin in 30 μM. In animal experiments, the mice were treated with t-AUCB (3 mg · kg^−1^ · d^−1^) by oral gavage or/and injected intraperitoneally with 20 mg/kg quercetin every day.

### Cell culture

Human glioblastoma cell lines U251 and U87 were provided by ATCC (American Type Culture Collection) as we used previously [15]. All cells were cultured in Dulbecco’s Modified Eagle’s Medium (DMEM) supplemented with 10 % fetal bovine serum (FBS) and 1 % penicillin and streptomycin (complete medium). Cells were maintained at 37 °C in a humidified atmosphere of 95 % air and 5 % CO_2_.

### Cell growth assay

Cell growth ability was exhibited by the cell counting kit-8 (CCK-8) from Dojindo Laboratories (Kumamoto, Japan). Cells were transplanted into a 96-well plate with density of 5000 cells/well and then treated differently. Cells were cultured in humidified incubator containing 5%CO2 and 95 % air. 48 hours later, culture medium in each well was discarded, and 100 μl fresh serum-free medium contained 10 μl CCK-8 solution was added into each well. After two hour incubation, the optical density value (absorbance) was recorded at 450 nm using an enzyme-linked immunosorbent assay plate reader (Bio-Rad Laboratories, Inc., Berkeley, CA, USA).

### Cell migration and invasion assays

Cell migration capacity was performed by wound healing assays. U251 cells were transplanted in 6-well plates. The scratch wounds were generated with a pipette tip. Cells were washed with PBS and incubated in a serum-free medium. 16 hours after scratching, wounds were fixed and photographic images were collected. Transwell assay was used to test cell invasion potency. U87 cells were transplanted in 24-well BioCoat Matrigel Invasion Chambers (8 μm pore size, Corning) with matrigel-coated in serum-free DMEM. Conditioned medium were cultured into the lower chambers like chemo-attractants. After incubation for 24 hours, the cells from upper surface of filters were scraped by a cotton swab, whereas matrigel were penetrated by other cells which adherent to the bottom of membrane. Methanol was used as fixed solution and 0.1 % crystal violet were used to stain.

### Cell-cycle analysis by flow cytometry

The cells were seeded in 10 cm culture dishes, then treated separately after adherence for 48 h at 37 °C in humidified incubator. Cells were collected followed by trypsinization, centrifuged (3500 rpm for 5 min), and washed twice with PBS. 1 ml 70 % ethanol was used as fixed solution, precipitated by centrifugation (3500 rpm for 5 min), and propidium iodide was used for staining nuclei. A total of 10,000 nuclei were analyzed in a FACSCalibur flow cytometer (BD Biosciences, San Jose, CA, USA).

### Apoptosis analysis by flow cytometry

For apoptosis analysis, the cells were seeded in 10 cm culture dishes, then treated separately after adherence for 48 h at 37 °C in humidified incubator. Cells were collected followed by trypsinization, centrifuged (3500 rpm for 5 min), and washed twice with PBS, the supernatant was discarded. The pellet was incubated away from light for 15 min at room temperature with Annexin V-fluorescein isothiocyanate (FITC) and propidium iodide (PI) before analysis with a FACSAria III flow cytometer (BD Biosciences, San Jose, CA, USA) according to the standard protocol.

### Caspase-3 activity assay

As we previously described [15], the activity of caspase-3 in cells was measured using caspase-3 activity assay kits from Millipore (Kankakee, IL, USA) following the manufacturer’s instructions. Absorbance was represented the level of caspase-3 activity and tested at 405 nm in a microtiter plate reader. In a word, cell samples were collected and resuspend in chilled 1 × Cell Lysis Buffer for 10 min, then centrifuged at 4 °C for 5 min in a microcentrifuge (10,000 × g). The supernatants were subsequently added into a 96-well plate and incubated with Ac-DEVD-pNA in the working solution containing caspase-3 substrate for 1 h at 37 °C. Fold-increase in caspase-3 activity was determined by comparing the absorbance from an apoptotic sample with an un-induced control after subtracting the background value reading from cell lysates and buffers. Each determination was performed in triplicate.

### RNA extraction and Quantitative real-time PCR

Total RNA was extracted from cells lysated by TRIzol reagent (Invitrogen, USA) according to the manufacturer’s instructions. The quality and quantity of the RNA purity were assessed by spectrophotometer and standard electrophoresis. cDNA was synthesized from 1 μg RNA and reverse transcribed using PrimerScript™ RT reagent Kit (Takara). The expression levels were quantified by Taqman™ Assay kit (Applied Biosystems, USA) in according with the manufacturer’s instructions. The sequences of primers for the COX-2 were as follows: forward: 5’- ATACCAAAACCGCATTGCCG-3’; reverse: 5’- TCTAACTCCGCAGCCATTTC-3’. Expression of GAPDH was performed as normalized. The sequences of primers for GAPDH were as follows: forward: 5’-GGAAGGTGAAGGTCGGAGTC-3’; reverse: 5’-GTCTTCTGGGTGGCAGTGAT-3’. Each reaction was performed by ABI 7500 fast RT- PCR system in triplicate. The expression of gene was defined based on the threshold cycle (Ct) value. The relative expression of the studied samples were assessed using the comparative delta-delta Ct method (TaqMan Relative Quantification Assay software), adjusted to GAPDH expression level.

### RNA interference

Cells were grown in 10-cm culture dish and transiently transfected with Hsp27 or COX-2 specific small interfering RNA (siRNA). The siRNA oligos of Hsp27 (sc-29350), COX-2 (sc-270376) and the negative control siRNAs were purchased from Santa Cruz Biotechnology (Dallas, TX, USA). Lipofectamine RNAiMAX Transfection Reagent (Invitrogen) was used for siRNA transfection according to the manufacturer’s protocol.

### Western blot analysis

Cell protein was lysates in ice-cold RIPA buffer (Beyotime Institute of Biotechnology, Shanghai, China) containing with Phenylmethanesulfonyl fluoride (PMSF) and protease inhibitor cocktail. The procedure of Western Blot was described as before [15]. The whole cell lysates were separated by SDS-polyacrylamide gel electrophoresis (SDS-PAGE) and transferred to a polyvinylidene fluoride membrane (Millipore Corporation, Bedford, MA, USA). All membranes were probed with primary antibodies after 4 °C overnight, and followed by incubation with secondary antibody. Proteins were visualized with chemiluminescence luminol reagents (Beyotime Institute of Biotechnology, Shanghai, China). Antibodies against β-actin (#3700), c-Myc (#9402), Cdc25A (#3652), Cyclin D1 (#2978), CDK4 (#12790), CDK6 (#3136), MCM3 (#4012), MCM7 (#3735), COX-2 (#12282), Hsp27 (#2402), p-Hsp27 (Ser78) (#2405) were purchased from Cell Signaling Technology (Beverly, MA, USA). Public software ImageJ (National Institutes of Health, USA) was used to quantify the densitometry of the immunoblotting bands.

### Tumor cell xenograft mouse models

Animal experiments protocols were approved by the Institutional Animal Committee of Jinling Hospital. The BALB/c nude mice (male at 5 to 6 weeks old) were obtain from Department of comparative medicine (Jinling Hospital, China) and maintained in specific pathogen-free (SPF) conditions. Approximately 1.0 × 10^6^ U87 cells were transplanted subcutaneously to develop a mouse xenograft model of human glioblastoma. Once tumor diameter reach to 2-4 mm, the treatment with quercetin or/and t-AUCB was administered. The length and width of the tumors were measured every other day using vernier caliper, and tumor volume was calculated (tumor volume = 1/2 × length × width^2^). The mice were treated with t-AUCB (3 mg · kg^−1^ · d^−1^) by oral gavage or/and injected intraperitoneally with 20 mg/kg quercetin every day. 14 days after treatment, all tumors retrieved from animals were measured, weighed and submitted for western blot analysis for the expression of Hsp27, p-Hsp27 and COX-2.

### Statistical analysis

All experiments were replicated in triplicate at least. The SPSS version 17.0 software (SPSS Inc., Chicago, IL, USA) was applied for statistical analysis. Comparisons between treated groups and vehicle control were performed using independent t test, and expressed as mean ± standard deviation (SD). *P* < 0.05 were considered statistically significant.

## Results

### t-AUCB inhibits cell proliferation, migration and invasion

The CCK-8 assay kit was used to test the cell proliferation. Human glioblastoma cell lines U251 and U87 were treated with vehicle control (DMSO), 10 μM, 50 μM, 100 μM, 150 μM, 200 μM, 300 μM or 400 μM t-AUCB for 48 h. As the results shown in Fig. 1, t-AUCB suppresses cell proliferation in a concentration-dependent manner since 100 μM (*P* < 0.05). 150 μM to 400 μM t-AUCB causes more efficient cell growth inhibition (*P* < 0.01). The IC50 of t-AUCB is 347.38 μM (to U87 cells) and 305.05 μM (to U251 cells).

**Fig. 2:**
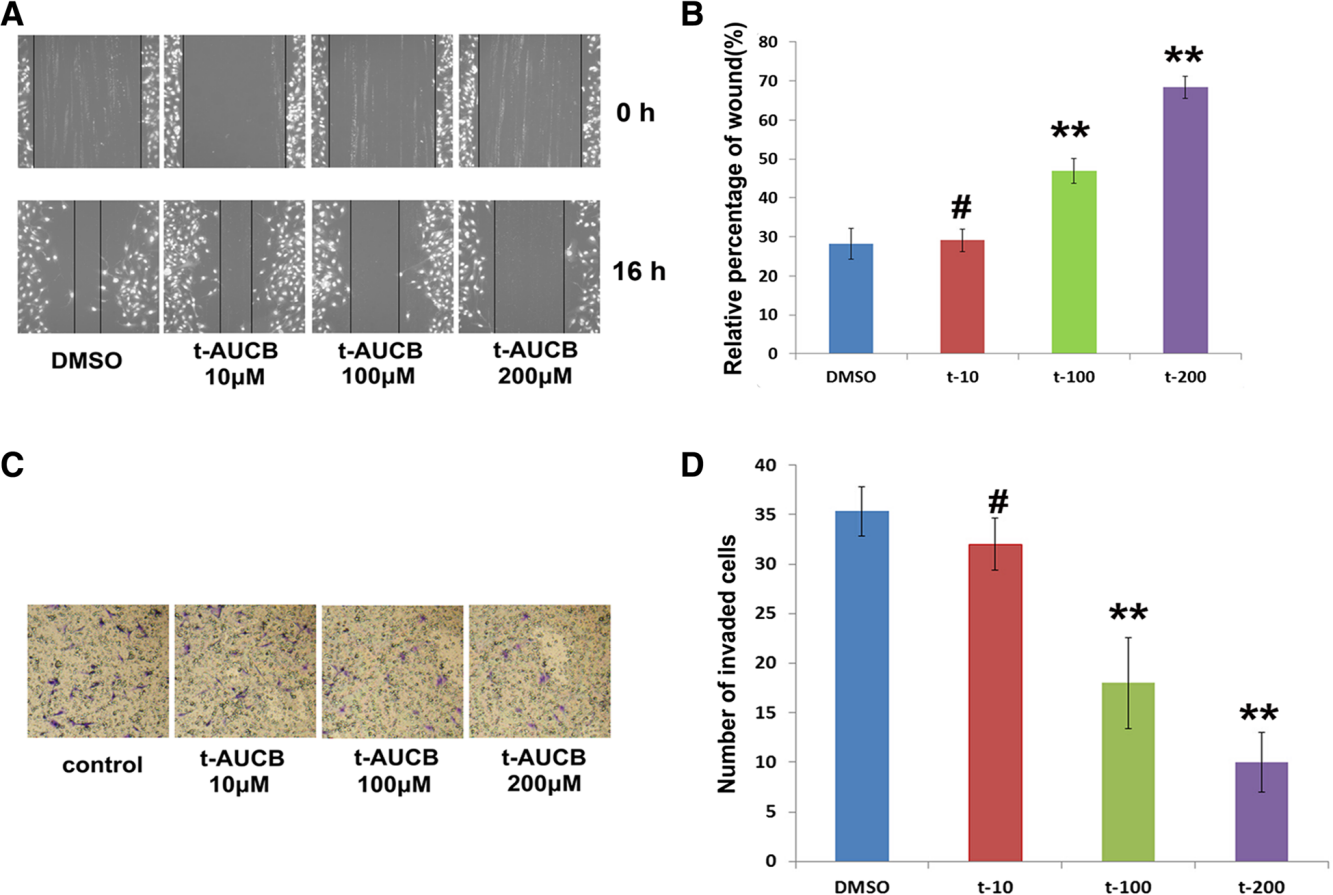
t-AUCB inhibits cell migration and invasion. **a**: wound healing analysis for U251 cells treated with vehicle control (DMSO), 10 μM, 100 μM or 200 μM t-AUCB. **b**: 100 μM or 200 μM t-AUCB treatment suppresses the scratched cell monolayer healing (**P < 0.01), whereas 10 μM t-AUCB has no effect (^#^ *P* > 0.05). **c**, **d**: matrigel-coated transwell chambers for cell invasion analysis, U87 cells were planted into the chambers and treated with vehicle control (DMSO), 10 μM, 100 μM or 200 μM t-AUCB. Cells treated with 100 μM or 200 μM t-AUCB showed a low level of penetration through the membranes compared with those treated with DMSO or 10 μM t-AUCB (**P < 0.01, ^#^ *P* > 0.05)

Cell migration was evaluated by wound healing analysis. U251 cells were planted and treated with vehicle control (DMSO), 10 μM, 100 μM or 200 μM t-AUCB for 16 h. 100 μM or 200 μM t-AUCB treatment suppresses the scratched cell monolayer healing, whereas 10 μM t-AUCB has no effect (Fig. 2a and b).

Cell invasion was analyzed using matrigel-coated transwell chambers. U87 cells were planted into the chambers and treated with vehicle control (DMSO), 10 μM, 100 μM or 200 μM t-AUCB for 24 h. Cells treated with 100 μM or 200 μM t-AUCB showed a low level of penetration through the membranes compared with those treated with DMSO or 10 μM t-AUCB (Fig. 2c and d).

### t-AUCB induces cell cycle G1 phase arrest

**Fig. 3:**
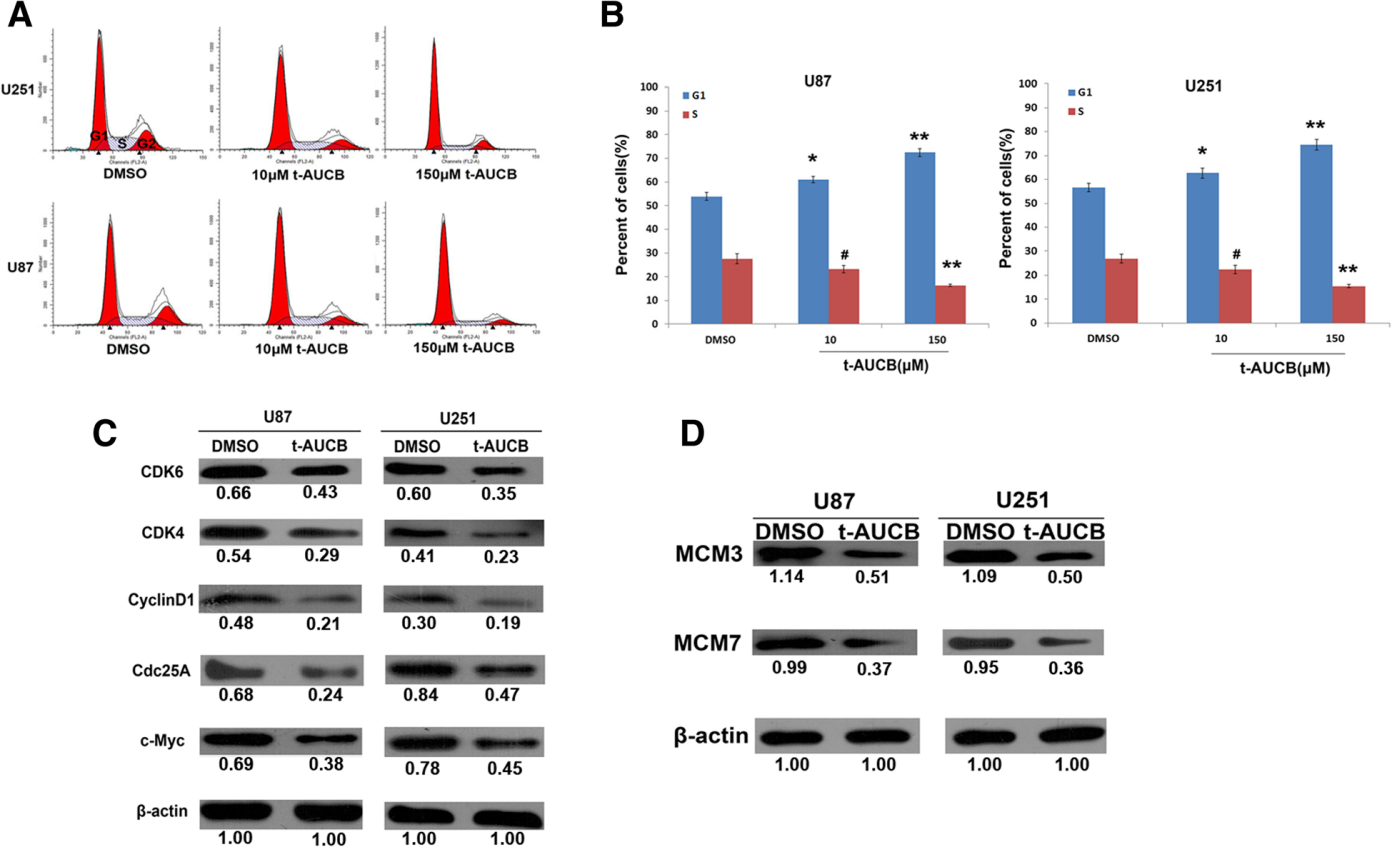
t-AUCB induces cell cycle G1 phase arrest. **a**, **b**: U251 and U87 cells were treated with DMSO (vehicle control), 10 μM or 150 μM t-AUCB for 48 h. 10 μM or 150 μM t-AUCB treated cells exhibited G1 phase proportion increase, 150 μM t-AUCB also induces S phase proportion decrease (**P < 0.01, *P < 0.05, ^#^ *P* > 0.05). **c**: The expression levels of c-Myc, Cdc25A, Cyclin D1, CDK4 and CDK6 were decreased in 150 μM treated cells. **d**: The expression levels of MCM3 and MCM7 were decreased in 150 μM treated cells. β-actin served as loading control. The protein intensity of the immunoblotting band was quantified by ImageJ and represented by the relative value compare with loading control (1.00)

For cell cycle assay, U251 and U87 cells were treated with DMSO (vehicle control), 10 μM or 150 μM t-AUCB for 48 h followed by flow cytometric analysis. 150 μM t-AUCB treated cells exhibited G1 phase proportion increase with S phase proportion decrease (Fig. 3a and b). Interestingly, 10 μM t-AUCB induces increase of G1 phase percentage, although it does not induce cell growth inhibition.

To further investigate how t-AUCB regulates cell cycle, we detected the regulators involved in G1/S phase cell cycle by western blot. In 150 μM t-AUCB treated cells, the expression levels of c-Myc, Cdc25A, Cyclin D1, CDK4 and CDK6, which play key roles in G1-S entry, were decreased (Fig. 3c). Moreover, DNA replication factors, MCM3 and MCM7, were found to decrease in t-AUCB treated cells (Fig. 3d).

### t-AUCB increases COX-2 expression by Hsp27 activation

**Fig. 4:**
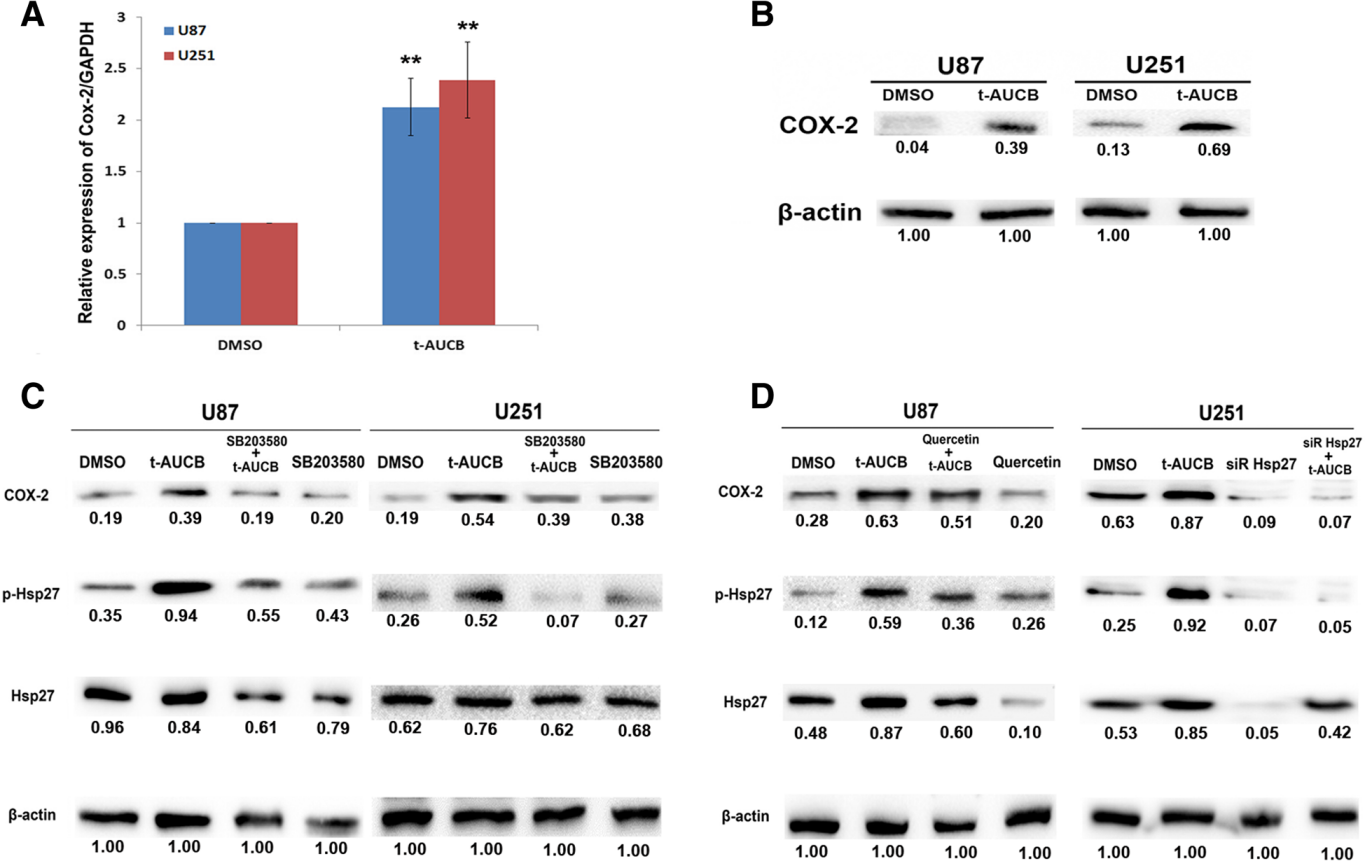
t-AUCB increases COX-2 expression by Hsp27 activation. 200 μM t-AUCB treatment increases COX-2 mRNA expression (**a**) (**P < 0.01) and protein expression (**b**). **c**: t-AUCB-induced COX-2 overexpression and Hsp27 phosphorylation was partially attenuated by SB203580. **d**: Hsp27 protein expression and its phosphorylation and COX-2 expression level were suppressed by quercetin or Hsp27 siRNA in cells without t-AUCB treatment. In cells treated with quercetin plus t-AUCB, decrease of COX-2 and Hsp27 expression and Hsp27 phosphorylation was partially reversed. β-actin served as loading control. The protein intensity of the immunoblotting band was quantified by ImageJ and represented by the relative value compare with loading control (1.00)

Our previous study has demonstrated that acquired Hsp27 activation confers resistance to t-AUCB in glioma cells [15]. Since COX-2 overexpression in gliomas is associated with poor prognosis [18], we hypothesized that COX-2 may participate in Hsp27 conferred resistance to t-AUCB. To test this hypothesis, we detected COX-2 mRNA level by qRT-PCR and its protein expression by western blot. Our data demonstrated that, t-AUCB treatment increases COX-2 expression in both mRNA and protein level (*P* < 0.01) (Fig. 4a and b). With the blockage of Hsp27 activation by p38 MAPK inhibitor SB203580, t-AUCB-induced COX-2 overexpression was partially attenuated (Fig. 4c), indicating the role of Hsp27 activation in COX-2 increase. To further investigate the effect of Hsp27 on COX-2 expression, cells were pretreated with Hsp27 inhibitor quercetin or Hsp27 siRNA followed by t-AUCB treatment. In cells without t-AUCB treatment, both Hsp27 protein expression and its phosphorylation level were suppressed by quercetin or Hsp27 siRNA, resulting in significant decrease of COX-2 expression (Fig. 4d). Interestingly, in cells treated with quercetin plus t-AUCB, decrease of COX-2 and Hsp27 expression and Hsp27 phosphorylation was partially reversed (Fig. 4d).

### Quercetin strengthens t-AUCB-induced cell growth inhibition by inhibition of Hsp27 and COX-2

**Fig. 5:**
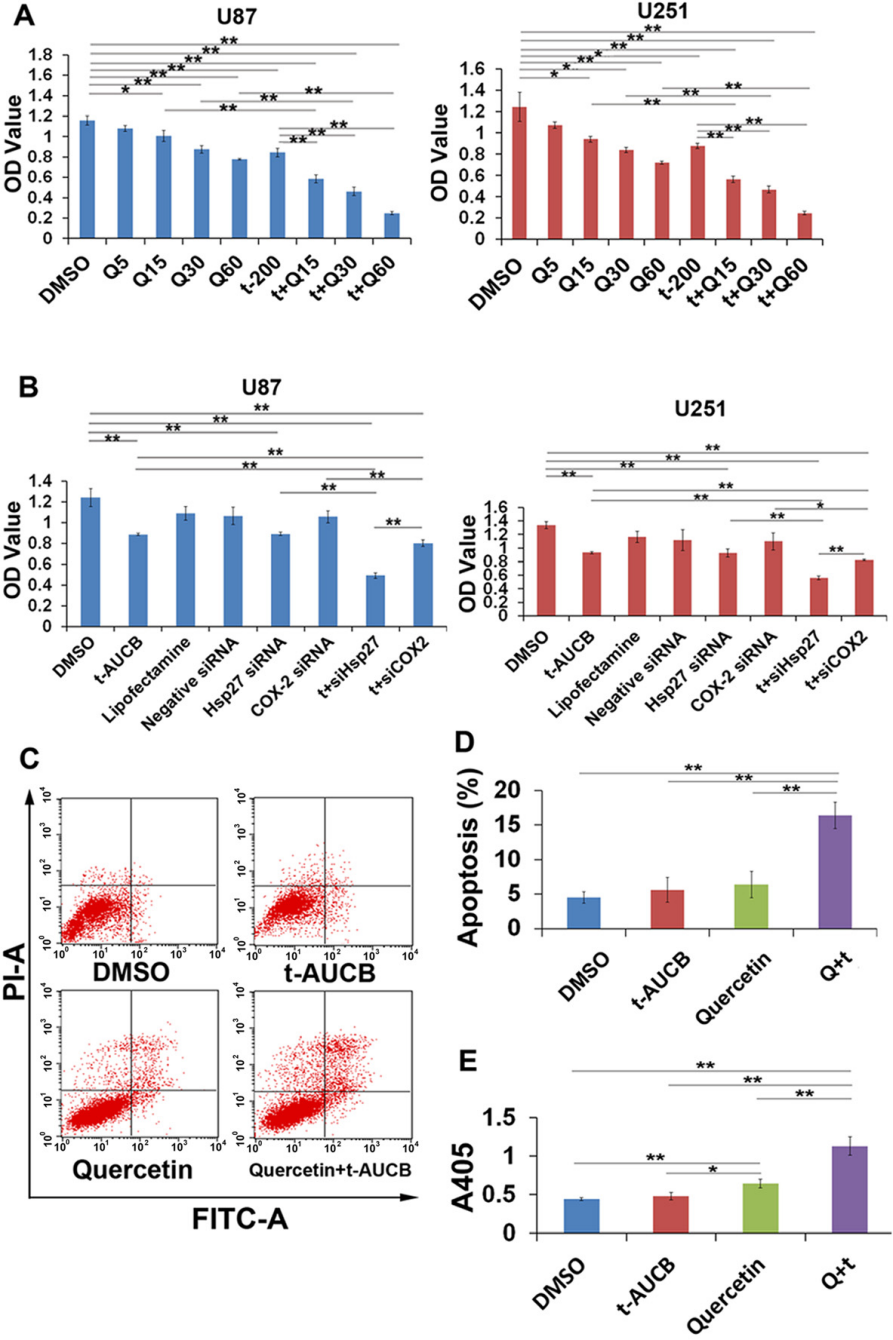
Quercetin strengthens t-AUCB-induced cell growth inhibition by inhibition of Hsp27 and COX-2. **a**: CCK-8 assay for cell viability. U87 and U251 cells were separately treated with vehicle control (DMSO), 5 μM, 15 μM, 30 μM, 60 μM quercetin, 200 μM t-AUCB, or 200 μM t-AUCB plus 15 μM, 30 μM or 60 μM quercetin for 48 h. The OD values represented the cell viability. Quercetin inhibits cell growth in concentration-depended manner since 15 μM, and strengthens t-AUCB induced cell growth inhibition. t-AUCB also strengthens quercetin-induced cell death (*P < 0.05, **P < 0.01). Q: Quercetin; t: t-AUCB. **b**: CCK-8 assay for cell viability. In U87 and U251 cells, knockdown of Hsp27 or COX-2 strengthens t-AUCB-induced cell growth inhibition (*P < 0.05, **P < 0.01). siHsp27: Hsp27 siRNA; siCOX-2: COX-2 siRNA; t: t-AUCB. **c**, **d**: Cell apoptosis analysis for U87 cells treated with vehicle control, 200 μM t-AUCB, 30 μM quercetin or 200 μM t-AUCB plus 30 μM quercetin for 48 h. Vehicle control treated cells the apoptosis proportion (Low Right and Upper Right section) was 4.51 ± 0.69 %. Cells treated with 200 μM t-AUCB exhibited no increase in apoptosis proportion of 5.62 ± 1.48 % (*P* > 0.05). Cells treated with 30 μM quercetin also exhibited no increase in apoptosis proportion of 6.35 ± 1.55 % (*P* > 0.05). For cells treated with 30 μM quercetin plus 200 μM t-AUCB, the apoptosis proportion was significantly increased into 16.38 ± 1.53 % (**P < 0.01). Q: Quercetin; t: t-AUCB. **e**: The caspase-3 activity assay for U87 cells treated with vehicle control, 200 μM t-AUCB, 30 μM quercetin or 200 μM t-AUCB plus 30 μM quercetin. OD value represented the caspase-3 activity (*P < 0.05, **P < 0.01). Q: Quercetin; t: t-AUCB

Since either inhibition of Hsp27 or COX-2 has been reported to facilitate glioma cell death [18–20], we then investigate whether quercetin, which inhibits both Hsp27 and COX-2 expression, could strengthens t-AUCB-induced cell growth inhibition. U87 and U251 cells were separately treated with vehicle control, 5 μM, 15 μM, 30 μM, 60 μM quercetin, 200 μM t-AUCB, or 200 μM t-AUCB plus 15 μM, 30 μM or 60 μM quercetin for 48 h. Cell growth was tested using CCK-8 kit. As the results shown in Fig. 5a, quercetin inhibits cell growth in concentration-depended manner since 15 μM, and strengthens t-AUCB induced cell growth inhibition. Moreover, the treatment of t-AUCB plus quercetin behaved more efficiently than quercetin alone (*P* < 0.01), indicating that t-AUCB can also strengthen quercetin-induced cell death. To further determine the role of Hsp27 or COX-2 in cell growth inhibition, synthetic siRNA was transfected into cells to knockdown expression of Hsp27 or COX-2. Given the downregulation of Hsp27 or COX-2, t-AUCB induces more intense cell death (*P* < 0.01) (Fig. 5b). Interestingly, it seemed that cells with knockdown of Hsp27 are more sensitive to t-AUCB than those with knockdown of COX-2 (*P* < 0.05). These results demonstrated that quercetin sensitizes cells to t-AUCB by downregulation of Hsp27 and COX-2.

Our previous study has reported that t-AUCB induces no significant cell apoptosis in U87 and U251 cells because of Hsp27 conferred resistance [15]. Herein, we investigated whether quercetin could reverse the apoptosis resistance by Hsp27 inhibition. U87 cells were treated with vehicle control, 200 μM t-AUCB, 30 μM quercetin or 200 μM t-AUCB plus 30 μM quercetin for 48 h. Cell apoptosis was analyzed using flow cytometer. As the results shown in Fig. 5c and d, in DMSO (vehicle control) treated cells the apoptosis proportion (Low Right and Upper Right section) was 4.51 ± 0.69 %. Cells treated with 200 μM t-AUCB exhibited no increase in apoptosis proportion of 5.62 ± 1.48 % (*P* > 0.05). Cells treated with 30 μM quercetin also exhibited no increase in apoptosis proportion of 6.35 ± 1.55 % (*P* > 0.05). For cells treated with 30 μM quercetin plus 200 μM t-AUCB, the apoptosis proportion was significantly increased into 16.38 ± 1.53 % (*P* < 0.01). The caspase-3 activity was also tested in different treated cells, as the data shown, t-AUCB plus quercetin significantly increases the activity of caspase-3 (*P* < 0.01) (Fig. 5e).

### Quercetin sensitizes glioblastoma to t-AUCB treatment in vivo

**Fig. 6:**
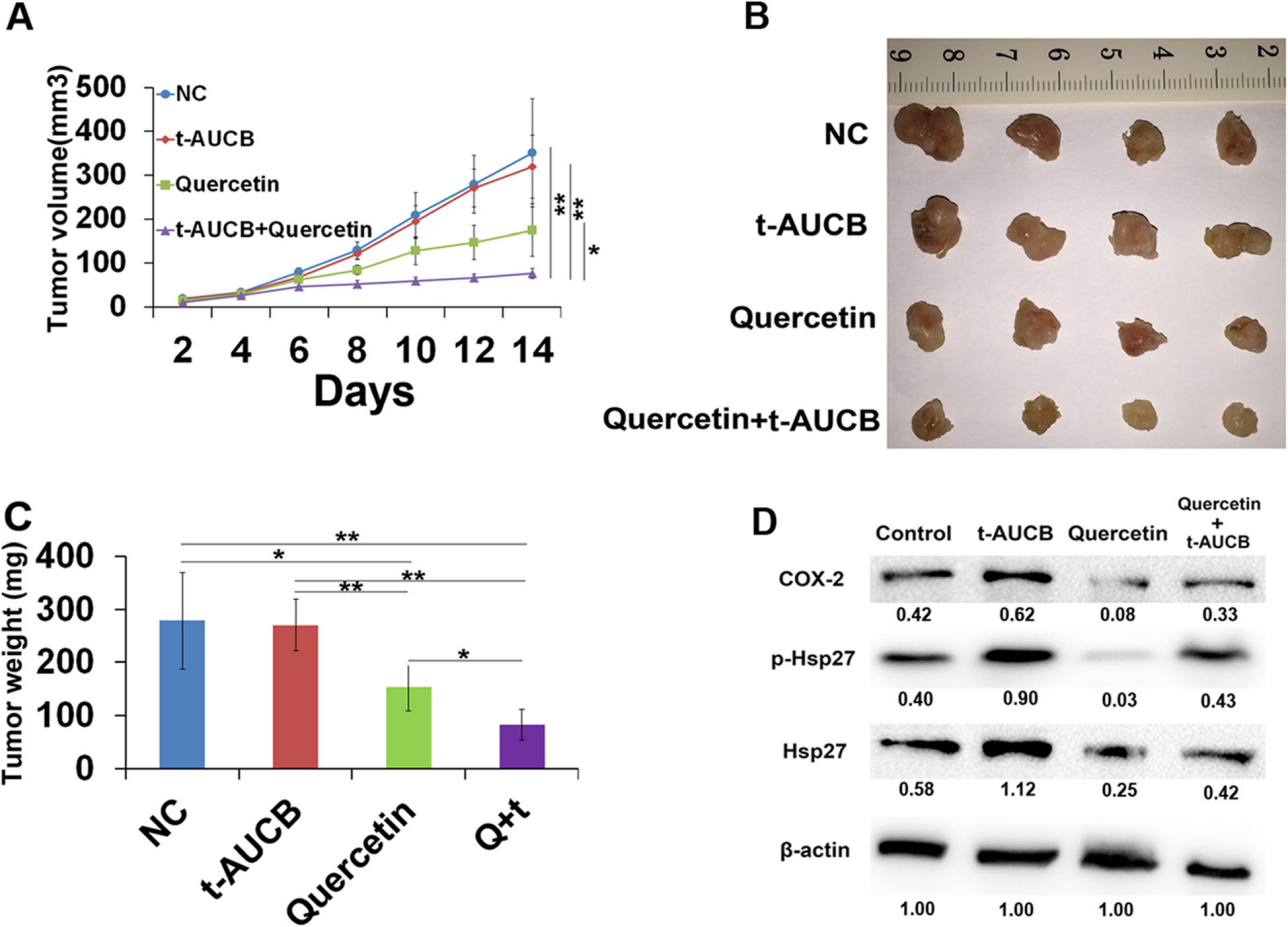
Quercetin sensitizes glioblastoma to t-AUCB treatment in vivo. **a**: Tumor bearing mice were differently treated and the tumor volumes were measured (*P < 0.05, **P < 0.01). **b**: xenograft tumors retrieved from animals. **c**: The weight of each tumor block was measured (*P < 0.05, **P < 0.01). **d**: The expression of Hsp27, p-Hsp27 and COX-2 in xenograft tumors by western blot. β-actin served as loading control. The protein intensity of the immunoblotting band was quantified by ImageJ and represented by the relative value compare with loading control (1.00)

To investigate the therapeutic effects of quercetin and t-AUCB on glioblastoma growth in vivo, we developed a mouse xenograft model of human glioblastoma by subcutaneously transplanting U87 cells in BALB/c nude mice. Once tumors grow to 2-4 mm in diameter, the treatment with quercetin or/and t-AUCB was administered as described in Materials and Methods section. The volume of xenograft tumor was measured and compared between different groups every two days up to 14 days. As the results shown in Fig. 6a and b, t-AUCB has no effect on tumor growth (*P* > 0.05). t-AUCB plus quercetin treatment exhibits significant tumor inhibition, compared with control group (*P* < 0.01), t-AUCB alone group (*P* < 0.01) or quercetin treatment group (*P* < 0.05). Moreover, the weight of each tumor block was measured. The data confirmed the results from tumor volume measurement (Fig. 6c). We then determined the expression of Hsp27, p-Hsp27 and COX-2 in xenograft tumors with different treatment by western blot. The levels of Hsp27 phosphorylation and COX-2 protein were elevated in t-AUCB treated tumors and sharply decreased in quercetin treated or quercetin plus t-AUCB treated tumors (Fig. 6d). These results suggested that t-AUCB induces no tumor growth inhibition in vivo, although it inhibits cell growth in vitro. Quercetin, a Hsp27 inhibitor, sensitizes glioblastoma to t-AUCB treatment and causes significant tumor inhibition in vivo.

## Discussion

We have demonstrated previously that acquired Hsp27 activation by p38 MAPK/MAPKAPK2/Hsp27 signaling cascade confers apoptosis-resistance to t-AUCB treatment in glioblastoma cells, although the cell proliferation inhibition and cell cycle G1 phase arrest induced by t-AUCB were significant [15]. Actually, in the present study, we further demonstrated that t-AUCB suppresses not only cell proliferation but also cell migration and invasion, as well as the expression of cell cycle G1/S transition factors and DNA replication factors. It seems that the effects of t-AUCB on glioblastoma cells are complicated with the concomitance of cytotoxicity and anti-cytotoxicity. Zhang et al. recently demonstrated that high-dose t-AUCB (10 mg · kg^−1^ · d^−1^) increases primary tumor growth and metastasis by stimulating tumor angiogenesis and VEGF levels, whereas low dose t-AUCB, 1 mg · kg^−1^ · d^−1^ or 3 mg · kg^−1^ · d^−1^, inhibits Lewis lung carcinoma metastasis or has no effect on primary tumor growth and metastasis, which makes the effects of t-AUCB on tumors more and more complicated [16]. sEHIs are considered to stimulate primary tumor growth and metastasis via EETs protection and following VEGF-dependent proangiogenic and protumorigenic effects [21, 22]. However, COX-2 inhibition has been shown to inhibit VEGF production, indicating that EETs-induced angiogenesis and tumorigenesis may be reduced or eliminated by inhibition of COX-2 [23]. Moreover, sEHIs may further sensitize the anti-inflammatory and antiangiogenic effects of COX-2 inhibition [13, 16]. Zhang et al. then demonstrated that the combination of t-AUCB and COX-2 inhibitor synergistically inhibits primary tumor growth and metastasis, although t-AUCB alone has no tumor inhibitory effect [16]. Previously, we have demonstrated similar results that combination of t-AUCB and inhibitor of Hsp27 phosphorylation effectively induced cell apoptosis and synergistically inhibits cell growth in vitro. Thus, we hypothesized that COX-2 and Hsp27 may interactively participate in conferring resistance to t-AUCB.

Our present data revealed that t-AUCB increases COX-2 expression by Hsp27 activation which is partially reversed by SB203580, a p38 MAPK inhibitor. Then, quercetin or Hsp27 specific siRNA was pretreated to inhibit Hsp27 expression before t-AUCB treatment. Decreased Hsp27 expression and activation resulted in significant decrease of COX-2 expression in cells without t-AUCB treatment, whereas Hsp27 expression and activation and COX-2 expression were partially suppressed by quercetin in t-AUCB treated cells. These results suggested that quercetin suppresses Hsp27 and COX-2 expression but t-AUCB partially reverse quercetin-induced Hsp27 and COX-2 suppression. However, combination of quercetin and t-AUCB induces significant cell apoptosis and more efficient cell growth inhibition than each alone (Fig. 5), although cells treated with quercetin alone show the least expression of Hsp27 and COX-2. It seems that the increased Hsp27 activation and COX-2 expression contribute to acquire resistance to t-AUCB treatment, and quercetin partially reverses the resistance by Hsp27 and COX-2 inhibition. The combination of quercetin and t-AUCB was also demonstrated to significantly inhibit glioblastoma growth in vivo from our data (Fig. 6). Although t-AUCB alone has no effect on xenograft glioblastoma, quercetin reverses the resistance of tumors to t-AUCB treatment and causes more efficient tumor growth inhibition than itself alone. Previously, combination of t-AUCB and Hsp27 phosphorylation inhibitor has been reported to synergistically inhibit glioblastoma cell growth in vitro by us [15], and combination of t-AUCB and COX-2 inhibitor has been shown to synergistically inhibit primary tumor growth by others [16]. Here we demonstrated for the first time that combination of t-AUCB and quercetin with co-inhibition of Hsp27 and COX-2 synergistically inhibits glioblastoma growth in vitro and in vivo.

Few literatures have mentioned the interaction of Hsp27 and COX-2 in tumors. Lasa, et al. demonstrated that p38 MAPK/MAPKAPK2/Hsp27 signaling cascade regulates COX-2 mRNA stability in HeLa cells [24]. They found that COX-2 mRNA is stabilized by the activation of p38 MAPK, which is blocked by SB203580, and that the effects of p38 MAPK are mediated by phosphorylation of Hsp27 [24]. Our present study demonstrated similar effect that COX-2 mRNA and protein expression is increased by Hsp27 activation. However, it is impossible to apply SB203580 or Hsp27 siRNA in clinical medication; thus, we decided to test the effects of Hsp27 inhibitor quercetin which had been studied in clinical trials on in vitro and in vivo system [25]. Quercetin, a bioflavonoid widely distributed in plants, is well known as one of the “nature agents” with antitumor effects [26, 27]. It has been demonstrated to inhibit Hsp27 and display antitumor activity in several tumor cells or cancer stem cells, such as lung cancer stem cells [28], breast cancer stem cells [29], oral cancer cells [30], hepatoma cells [31], human Ewing’s tumor cells [32], prostate cancer cells [27], as well as glioblastoma cells [33]. Our data demonstrated a novel supplementation that quercetin inhibits both Hsp27 and COX-2 and eliminates the resistance to t-AUCB in glioblastoma.

## Conclusions

Our study demonstrates that t-AUCB inhibits cell proliferation, migration and invasion and induces cell cycle G1 phase arrest in vitro; increased Hsp27 activation and following COX-2 overexpression confer resistance to t-AUCB treatment in glioblastoma in vitro and in vivo; quercetin sensitizes glioblastoma to t-AUCB by dual inhibition of Hsp27 and COX-2. Combination of t-AUCB and quercetin may be a potential approach to treating glioblastoma.

## Abbreviations

EETs: Epoxyeicosatrienoic acids
ARA: arachidonic acid
sEHIs: soluble epoxide hydrolase inhibitors
DHETs: dihydroxyeicosatrienoic acids
Hsp27: Heat shock protein 27
COX2: cyclooxygenase 2
DMSO: dimethyl sulfoxide
DMEM: Dulbecco’s Modified Eagle’s Medium
FBS: fetal bovine serum
CCK-8: cell counting kit-8
FITC: fluorescein isothiocyanate
PI: propidium iodide
